# Protective role of STVNa in myocardial ischemia reperfusion injury by inhibiting mitochondrial fission

**DOI:** 10.18632/oncotarget.22969

**Published:** 2017-12-05

**Authors:** Xiaoou Sun, Yingying Yang, Yanxiang Xie, Xingjuan Shi, Lijie Huang, Wen Tan

**Affiliations:** ^1^ Institute of Biomedical and Pharmaceutical Sciences, Guangdong University of Technology, Guangzhou 510006, China; ^2^ School of Bioscience and Bioengineering, South China University of Technology, Guangzhou 510006, China; ^3^ Key Laboratory of Developmental Genes and Human Disease, Institute of Life Sciences, Southeast University, Nanjing 210096, China

**Keywords:** STVNa, mitochondrial, ischemia reperfusion injury, cardioprotective

## Abstract

It has been reported that isosteviol, a widely known sweeteners, can protect against myocardial ischemia-reperfusion (IR) injury in isolated guinea pig heart. Here, we aim to confirm the cardioprotective effect of its sodium salt, isosteviol sodium (STVNa), against IR injury and its potential molecular mechanism in H9c2 cardiomyocytes. STVNa significantly improved cell viability, restored mitochondrial membrane potential, decreased cellular reactive oxygen species generation, and inhibited cell apoptosis. Furthermore, STVNa treatment changed the morphology of mitochondria from fragmented, discontinuous forms to normal elongated, tubular forms. Cyto-immunofluorescence and western blot analysis revealed that STVNa inhibited mitochondrial fission proteins dynamin-related protein 1 (Drp1), and mitochondrial fission 1 (Fis1), thus plays a key role in cardioprotection. These findings, for the first time, suggest that STVNa can protect against myocardial IR injury through reverse mitochondrial fission.

## INTRODUCTION

Isosteviol, is the acid hydrolysate product of stevioside, a widely known sweeteners. In recent years, a number of studies have investigated the anti-hyperglycemic [1], anti-hypertensive [2], anti-inflammatory [3] and anti-tumor effects of isosteviol [4]. The cardioprotective effect of isosteviol in ischemia-reperfusion (IR) injury in isolated guinea pig heart has also been reported [5, 6]. Although potassium channels and calcium channels have been reported to be involved in the action of isosteviol, the potential cardioprotective mechanism of isosteviol is not yet clear [7].

Myocardial IR injury is a major cause of death and disability worldwide. The pathophysiology of myocardial reperfusion injury can induce arrhythmias, myocardial stunning, oxidative stress, intracellular Ca^2+^ overload, and mitochondrial permeability transition pore (mPTP) opening [8]. Processes involved in myocardial cell IR-injury include inflammation, excitotoxicity, mitochondrial dysfunction, and oxidative stress, and cell apoptosis [8, 9]. Mitochondria are important regulators of cell growth, and are highly dynamic organelles involved in fusion and fission in response to ischemia or other types of oxidative stress [9]. The morphology of normal mitochondria is tubular, elongated and interconnected. However, when undergoing IR-injury the mitochondrial permeability transition pore opens, mitochondria dynamic balance would be lost, lead to changes in morphology and the mitochondria are fragmented and discontinuous. It has been shown that dynamin-related protein 1 (Drp1), and mitochondrial fission 1 (Fis1) play key roles in this process [10]. Changes in mitochondria morphology also have an impact on mitochondrial membrane potential (ψ_m_), impair the redox buffer system and induce reactive oxygen species (ROS) production [11]. Mitochondrial fission proteins affect the function of the organelle and eventually integrated cellular signaling cascades, including apoptosis. In the present study, we found that isosteviol sodium (STVNa), the sodium salt of isosteviol protects H9c2 cardiomyocytes against IR injury through inhibition of the mitochondrial fission pathway.

## RESULTS

### Effect of STVNa on H9c2 cell viability following different ischemia times

**Figure 1:**
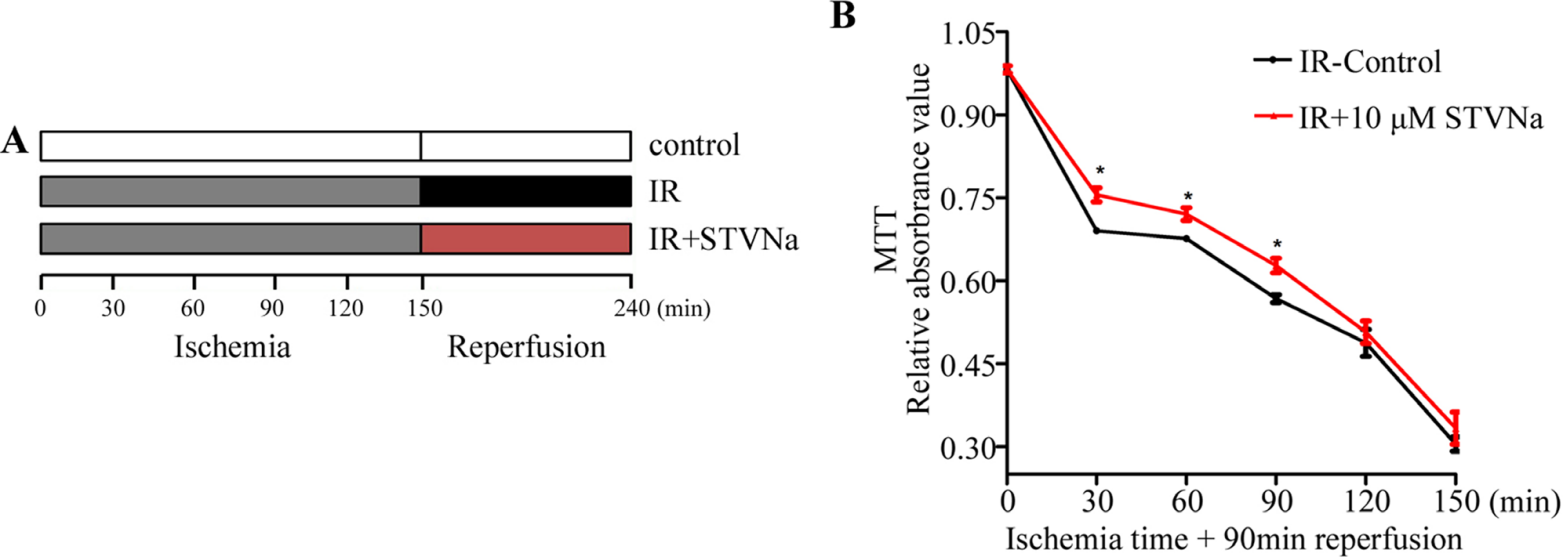
Effect of IR on H9c2 cell viability and the protective effect of STVNa in different ischemia conditions (**A**) The protocol used to investigate the appropriate ischemia time and the effect of STVNa on cell viability in different ischemia conditions. (**B**) H9c2 cells were subjected to 0, 30, 60, 90, 120, and 150 min ischemia followed by reperfusion with or without 10 μM STVNa for 90 min. Cell viability was assessed in the different treatment groups by the MTT assay. Data are shown as mean ± S.E.M in four independent experiments. ^*^P < 0.05 vs. IR.

H9c2 cells were subjected to 0, 30, 60, 90, 120, and 150 min ischemia followed by reperfusion with or without STVNa for 90 min. The results showed that with extended ischemia time, cell viability gradually decreased to 30.4% ± 1.3% compared to the control. At that time IR injury was serious and unable to recover. However, following ischemia for 30, 60, and 90 min, 10 μM STVNa showed a significant protective effect (*P* < 0.05, Figure 1B). Therefore, we chose an ischemia time of 90 min for the following experiment as cell viability was 69.6% ± 1.3%.

### STVNa restored mitochondrial membrane potential (Δψ) during IR

**Figure 2:**
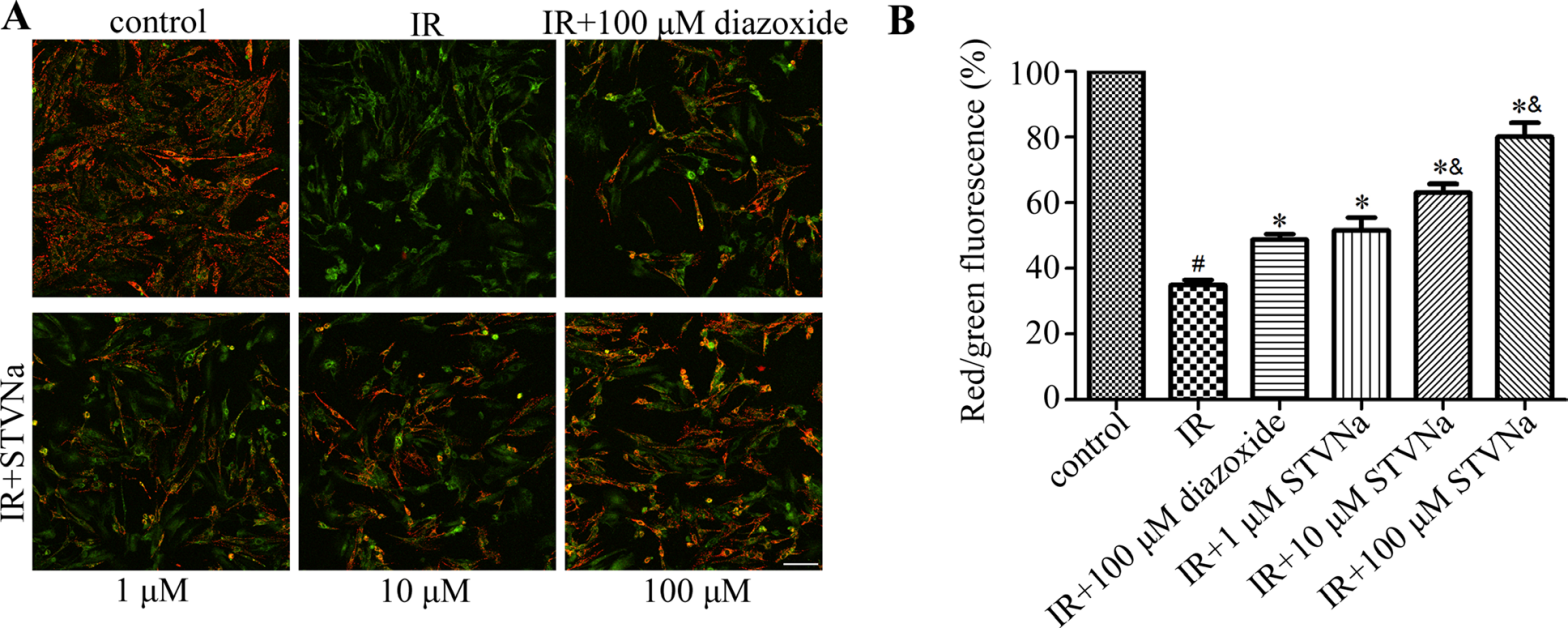
Effect of STVNa on mitochondrial membrane potential after IR (**A**) Confocal images of mitochondrial potential following JC-1 staining. Scalebar: 100 μm. (**B**) Graph of red-to-green (R/G) fluorescence intensity. Diazixide was used as a positive control. Images representative of three individual experiments. Data are expressed as percentages of the control level. All values are expressed as means ± S.E.M. ^#^*P* < 0.05 vs. control; ^*^P < 0.05 vs. IR; ^&^*P* < 0.05 vs. 100 μM diazoxide.

Decreased cell viability is generally associated with a disturbance in mitochondrial function. The dissipation of Δψ is an indication of failing mitochondria. We assessed the effect of STVNa on Δψ using the membrane sensitive dye JC-1. As shown in Figure 2, 90 min of ischemia followed by 90 min of reperfusion resulted in a marked decrease in Δψ (R/G ratio: 0.328 ± 0.006 vs 0.944 ± 0.03 in the control group). Cells treated with 1, 10, and 100 μM STVNa partially recovered Δψ in a dose-dependent manner (*P* < 0.05). Diazoxide was used as a positive control (*P* < 0.05). Furthermore, 10 and 100 μM STVNa had a better effect than 100 μM diazoxide in maintaining Δψ (*P* < 0.05).

### STVNa decreased IR-induced intracellular ROS production

**Figure 3:**
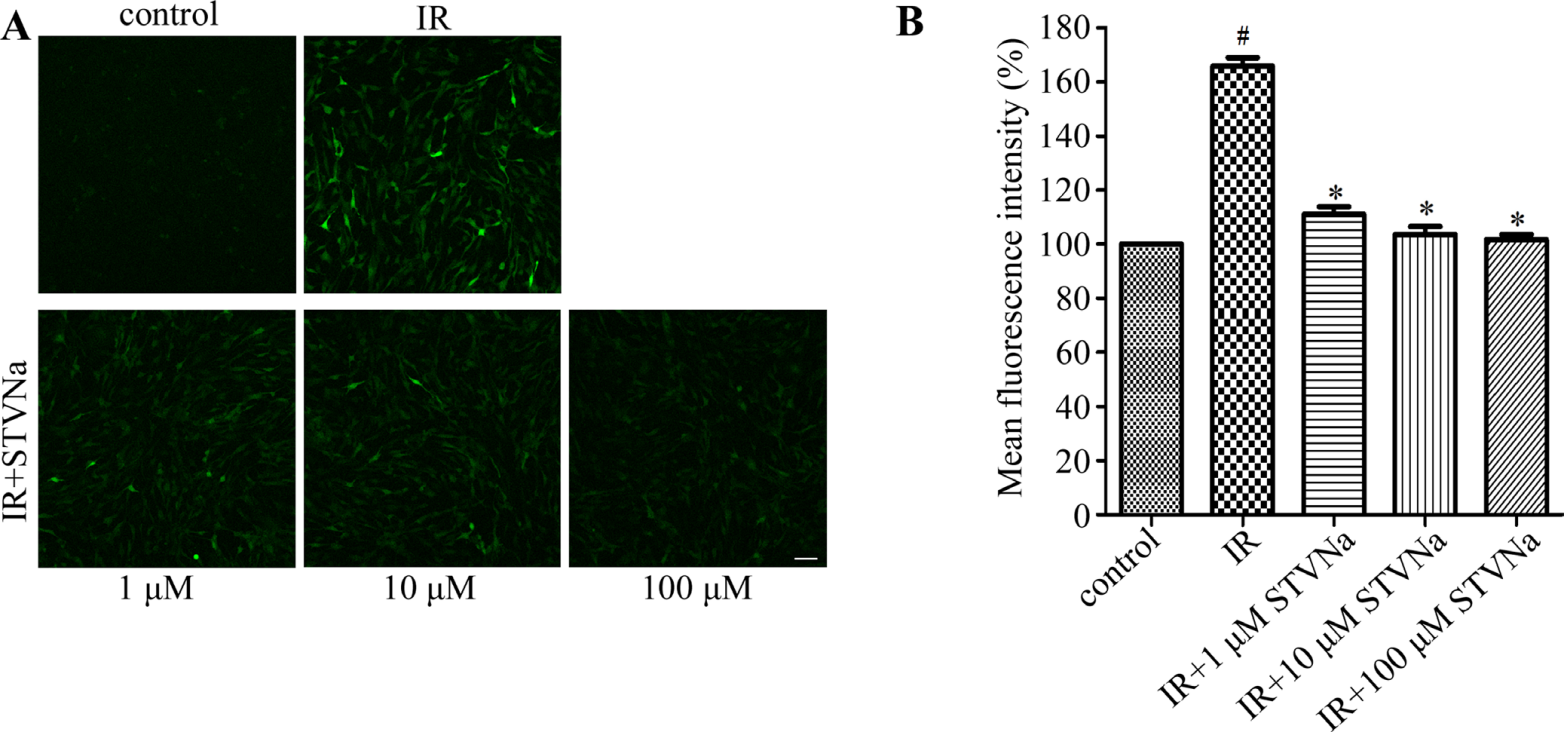
STVNa decreased IR-induced intracellular ROS production (**A**) Confocal images of ROS. Intracellular ROS production was measured by DCFH-DA staining. Scalebar: 100 μm. (**B**) Graph of ROS levels. Images representative of three individual experiments. Data are expressed as percentages of the control level. All values are expressed as means ± S.E.M. ^#^*P* < 0.05 vs. control; ^*^P < 0.05 vs. IR.

Oxidative stress is the primary contributor in IR injury. It can induce modifications in mitochondrial proteins, DNA and lipids, which inhibit energy production and contractile function, eventually leading to cell apoptosis [12]. To determine whether STVNa inhibited oxidative stress, intracellular ROS production was measured by DCFH-DA staining. Figure 3 shows that IR induced a burst in ROS production (mean fluorescence intensity: 1.688 ± 0.024 vs 1.030 ± 0.013 in the control). STVNa (1, 10 and 100 μM) significantly reduced ROS accumulation (*P* < 0.05).

### STVNa inhibited IR-induced cell apoptosis

**Figure 4:**
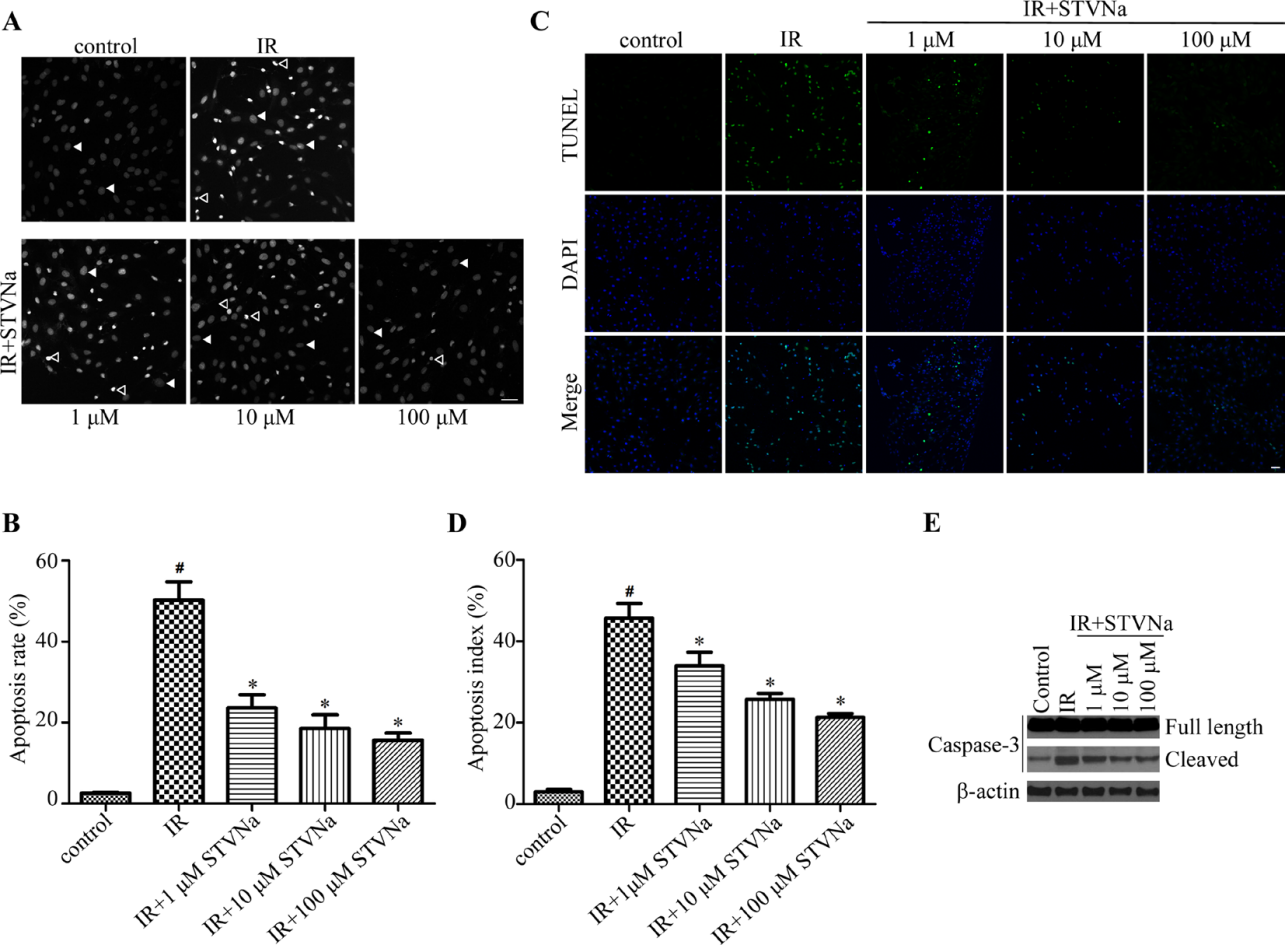
STVNa inhibited IR-induced cell apoptosis (**A**) Confocal images of apoptosis associated with nuclear condensation in H9c2 cardiomyocytes. H9c2 cells from different treatment groups were evaluated by the DNA-binding fluorescent dye DAPI and images were then taken under the fluorescence microscope. The arrowhead and open arrowhead indicate respectively. Scalebar: 100 μm. (**B**) Experiments were performed as in (A), and the ratio of NCI cells in each group was counted. *n* = 100 per group. (**C**) The fluorescence images of TUNEL staining. H9c2 cells from different treatment groups were evaluated by TUNEL (green) and DNA dye DAPI (blue) and images were then taken under the fluorescence microscope. (**D**) Experiments were performed as in (C), and the percent of apoptosis cells were decided by TUNEL positive cells (green) to the total DAPI staining cells (blue). The data were then normalized to the control group. All values are expressed as means ± S.E.M, *n* = 100 per group. ^#^*P* < 0.05 vs. control; ^*^P < 0.05 vs. IR. (**E**) The activation of caspase-3 in different treatment groups was analyzed by immunoblot analysis.

To determine the protective effect of STVNa on cell apoptosis during H9c2 cell IR injury. Cells from different treatment groups were evaluated by the DNA-binding fluorescent dye DAPI and TUNEL. The morphology of a normal cell nucleus is large and uniform, however, condensation and enhanced fluorescence intensity were seen in apoptotic cells (Figure 4A). Quantitation of the extent of NCI using Image-Pro Plus analysis revealed that the proportion of NCI in the IR group was 50.22% ± 4.6% and decreased to 23.64% ± 3.2% (*P* < 0.05), 18.52 % ± 3.4 % (*P* < 0.05), and 15.65% ± 1.8% (*P* < 0.05) following treatment with 1, 10, and 100 μM STVNa, respectively. TUNEL method was also used to examine the occurrence of cell apoptosis (Figure 4C, 4D). The percent of TUNEL positive cells was markedly increased in IR group compared to control, while the number of apoptosis cells in 10 μM STVNa treatment group was obviously reduced (*P* < 0.05). We further examined caspase-3 activation of cells with different treatment, and found that STVNa reduces the effect of IR-injury on caspase-3 activation (Figure 4E).

### Effect of STVNa on mitochondrial morphology in confocal images

**Figure 5:**
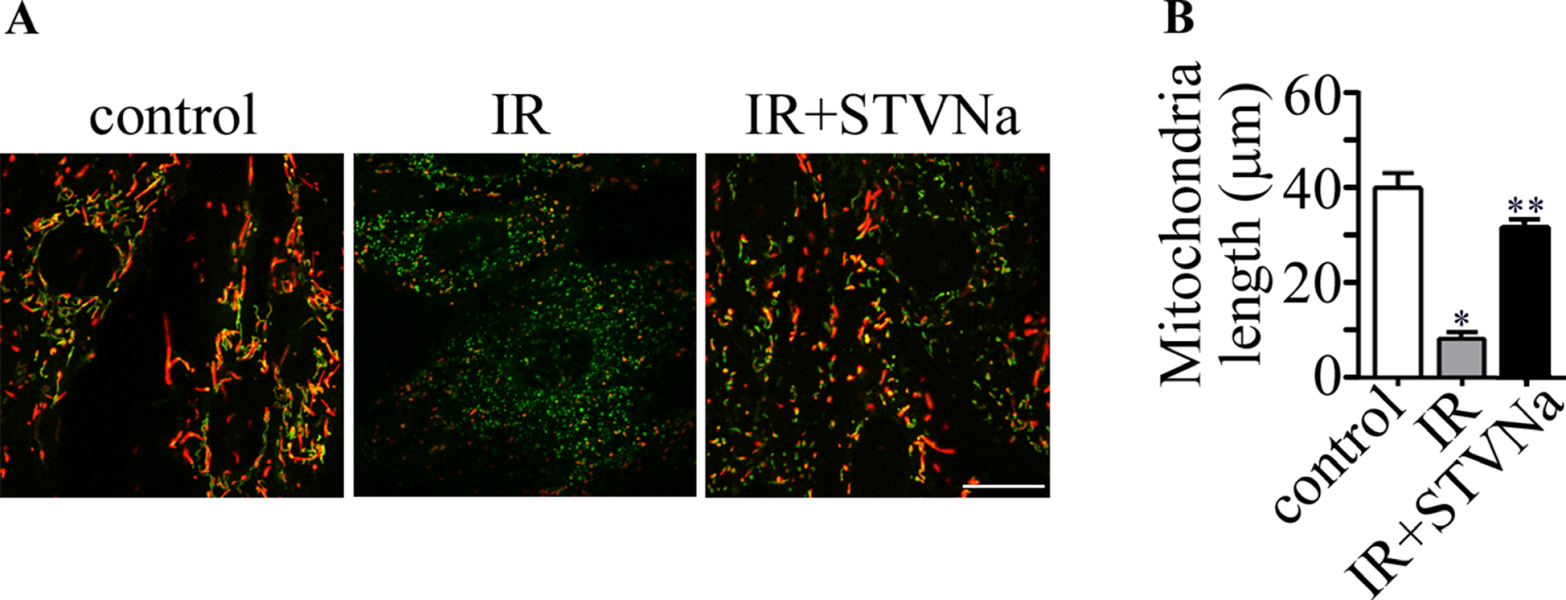
Changes in mitochondrial morphology during IR (**A**) JC-1 staining shows the tubular mitochondrial morphology in H9c2 cells under normal conditions. Small and fragmented mitochondria were evident in cells undergoing IR. STVNa (10 μM) recovered mitochondrial morphology to some extent. Scalebar: 100 μm. (**B**) Experiments were performed as in (A), and the length of mitochondria were measured. The data were then normalized to the control group. All values are expressed as means ± S.E.M, *n* = 40 per group ^*^P < 0.05; ^**^P < 0.01.

We examined the changes in mitochondrial morphology during IR injury. As shown in Figure 5, the morphology of mitochondria became fragmented or discontinuous after IR. However, this effect was largely inhibited by 10 μM STVNa. Taken together, pharmacological treatment with STVNa inhibited abnormal mitochondrial fission and reduced cell death.

### STVNa inhibits the expression of proteins related to mitochondrial morphology

**Figure 6:**
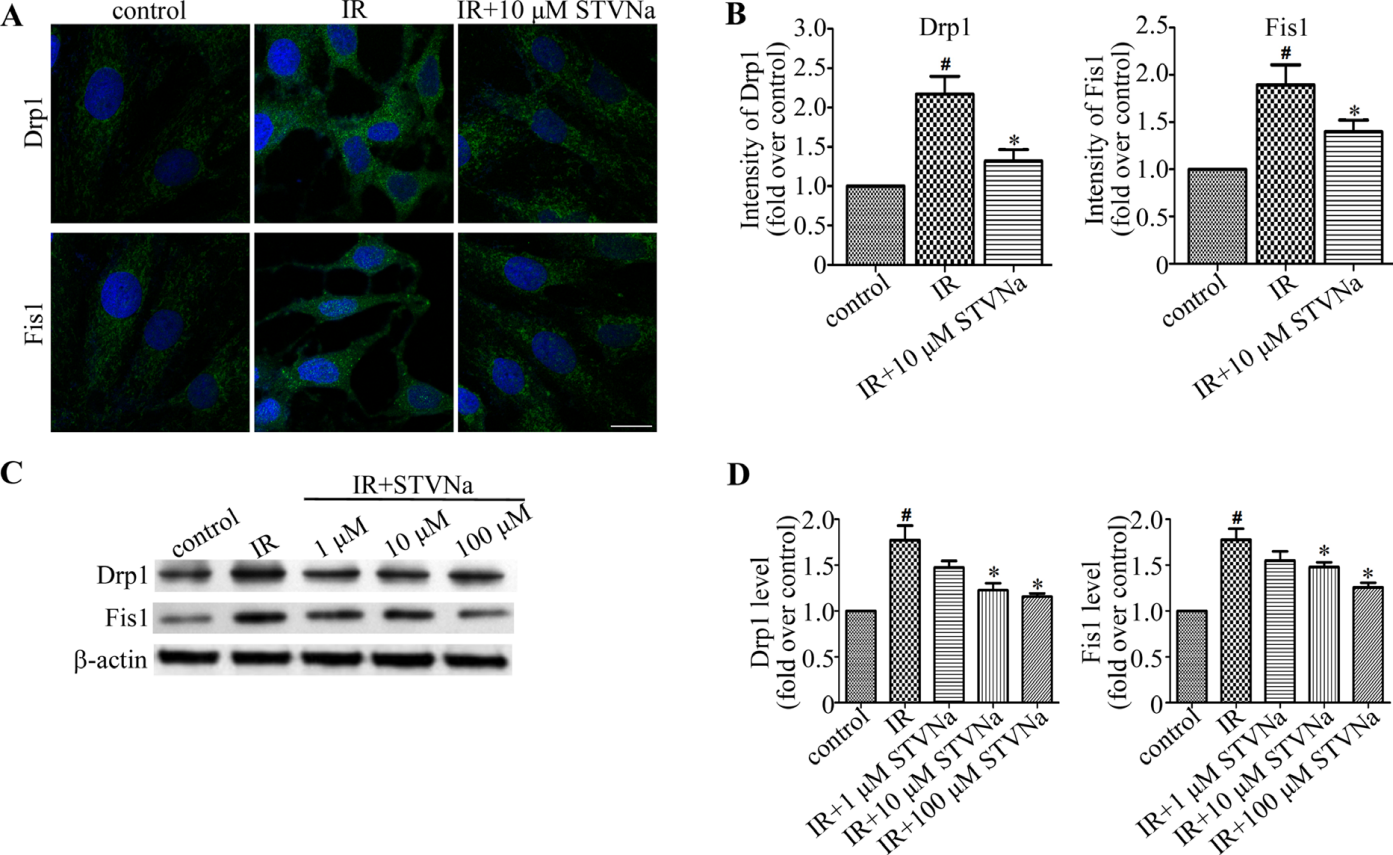
STVNa inhibited mitochondrial fission proteins Drp1 and Fis1 (**A**) H9c2 cells in each group were stained with antibodies against Drp1 and Fis1 (green) and the DNA dye DAPI (blue), and images were then taken under the fluorescence microscope. Scalebar: 50 μm. (**B**) Experiments were performed as in (A), and the levels of Drp1 and Fis1 were analyzed by measuring the fluorescence intensity in each group. The data were then normalized to the control group. (**C**) The levels of Drp1, Fis1 and actin were examined by immunoblotting with antibodies against Drp1, Fis1 and b-actin. (**D**) Experiments were performed as in (C), and the level of Drp1 and Fis1 was analyzed. Protein levels are relative to the control group. All values are expressed as means ± S.E.M in four independent experiments. ^#^*P* < 0.05 vs. control; ^*^P < 0.05 vs. IR.

To determine the underlying molecular mechanism of STVNa in the inhibition of mitochondrial fragmentation in myocardial IR injury, we assessed the expression of the mitochondrial fission related proteins, Drp1 and Fis1, by immunofluorescence and western blot analysis. As shown in Figure 6, compared to the control cells, the fluorescence intensity of Drp1 and Fis1 in IR cells was increased (*P* < 0.05). Western blot analysis also revealed that the expression of Drp1 and Fis1 proteins was up-regulated (*P* < 0.05). However, when treated with STVNa during reperfusion, the fluorescence intensity and protein expression of Drp1 and Fis1 were significantly decreased (*P* < 0.05). Thus, we suggest that STVNa inhibits IR-induced cardiomyocyte apoptosis through regulating the expression of mitochondrial fission proteins, which play key roles in myocardial IR injury.

## DISCUSSION

Mitochondria, are the energy source in cells, and are highly dynamic organelles. They can change in number and morphology within a cell during development in response to various stimulants [13]. In myocardial IR injury, mitochondrial damage including Ca^2+^ overload, ROS overproduction, and the opening of mPTP plays a key role in cardiomyocyte apoptosis [14]. There are a number of proteins which participate in mitochondrial fusion and fission, of which Drp1 is the mostly frequently studied [15]. Fis1 is thought to act as a mitochondrial receptor for Drp1 [16]. In response to various stimulants, Drp1 translocates from the cytosol to the mitochondrion and wraps around the mitochondrion to induce fission by binding to its mitochondrial adaptors such as Fis1 [17]. It has been reported that the inhibition of mitochondrial fission can protect the heart against myocardial IR injury both *in vivo* and *in vitro* [18, 19]. This may be a new target for IR therapy [20]. In the present study, the expression of Drp1 and Fis1 in the IR group was significantly increased accompanied by cell death. Conversely, pharmacological inhibition with STVNa improved mitochondrial morphology and reduced cell death. These changes at the molecular level also corresponded to the dynamic transformation of mitochondria morphology.

Previous studies have revealed that inhibition of Drp1 increases cell survival, decreases IR-associated calcium and ROS, and preserves mitochondrial oxygen consumption and morphology [18]. Impairment of mitochondrial integrity results in ROS production, DNA damage and activation of cell death [21]. In the current study, the rat ventricular H9c2 cell line was used. This cell line has been used extensively in studies investigating signal transduction mechanisms in cardiomyocytes as it retains properties of the signaling pathways of cardiomyocytes. It is also suitable for *in vivo* models to simulate IR injury due to its high sensitivity [22]. In our experiment, when cell viability was approximately 50% these cells were used as an IR model. STVNa significantly improved cell survival rate which was determined using the MTT assay. In subsequent experiments, the myocardial cells were subjected to 90 min of ischemia followed by 90 min of reperfusion. In ischemic heart disease and other cardiomyopathies, mitochondrial dysfunction is a key factor in inducing cell death. The inhibition of mPTP opening and mitochondrial antioxidants are two main therapeutic targets [23]. mPTP opening results in mitochondrial swelling and the depolarization of Δψ [24]. Oxidative stress causes cell damage due to modifications in mitochondrial proteins, DNA, and lipids [25]. It has been proved that STVNa can both preserve Δψ and significantly decrease reperfusion-induced ROS overproduction in a dose-dependent manner. Furthermore, compared to the selective mitochondrial ATP-sensitive potassium channel opener diazoxide, which has been reported to protect cardiac mitochondria [26, 27], STVNa has a better protective effect.

The final event in IR is cell apoptosis. Apoptosis, which is programmed cell death, is characterized by cell shrinkage and pyknosis. It is the result of chromatin condensation [28]. The cell nucleus is shrunken and irregular during apoptosis which can be detected by nucleic acid staining with DAPI due to its high affinity for DNA. The present study shows that treatment with STVNa inhibits cell apoptosis by adjusting a series of upstream signal pathways.

## MATERIALS AND METHODS

### Chemicals and antibodies

STVNA was provided by the Chemical Development Laboratories of Key Biological Pharmaceutical Company (Dongguan, China). The JC-1 and 2’,7’-dichlorofluorescin diacetate (DCFH-DA) were purchased from Life Technology. Diazoxide, Terminal Deoxynucleotidyl Transferase-mediated dUTP Nick End Labelling (TUNEL) and 3-(4, 5-dimethylthiazolyl-2)-2, 5-diphenyl-2-H-tetrazolium bromide (MTT) were from Sigma-Aldrich. 4’,6-diamidino-2-phenylindole (DAPI), and antibodies against Drp1, Fis1, Caspase-3, Cleaved Caspase-3 and b-actin were purchased from Abcam. Horseradish peroxidase-conjugated secondary antibodies were purchased from Santa Cruz Biotechnology, and fluorescein-and rhodamine-conjugated secondary antibodies were from Jackson ImmunoResearch Laboratories.

### Cell culture and IR injury model

The H9c2 cells were obtained from the Type Culture Collection of the Chinese Academy of Sciences (Shanghai, China) and cultured according to the manuals. IR injury was induced as previously reported. Briefly, H9c2 cells were seeded in a six-well plate containing 2 × 10^5^ cells/well. After adherence for 24 h, the medium was replaced with ischemia buffer (137 mM NaCl, 15.8 mM KCl, 0.49 mM MgCl_2_, 0.9 mM CaCl_2_, 4 mM HEPES, 10 mM 2-deoxyglucose, 20 mM sodium lactate and 1 mM sodium dithionite, adjusted to pH 6.5) to mimic ischemia *in vitro*. The cells were then incubated in a hypoxia chamber containing 94% N_2_, 1% O_2_, and 5% CO_2_ for a period of time. At the end of ischemia, the buffer was discarded and replaced with fresh DMEM buffer. The cells were incubated for a further 90 min in a normal environment. In the drug treatment groups, 1, 10, and 100 μM STVNa were added to the reperfusion buffer at the onset of reperfusion (Figure 1A).

### Cell viability assay

H9c2 cells were seeded at a density of 1 × 10^4^/well in a 96-well plate and the cell IR injury model was established as described above. After adherence for 24 h, the cells were incubated in a ischemia buffer for 90 min. At the end of ischemia, the cells were incubated for a further 90 min in a normal environment. H9c2 cells were subjected to 0, 30, 60, 90, 120, and 150 min ischemia followed by reperfusion with or without STVNa for 90 min. Cell viability was assessed in the different treatment groups by the MTT assay then. The MTT stock solution (5 mg/mL) was diluted with DMEM to a final concentration of 0.5 mg/mL and 100 μL was added to each well. Following incubation for 4 h at 37°C, the supernatants were discarded and 150 μL/well DMSO was used to dissolve the formazan crystals. The optical density was measured at 490 nm using a microplate reader.

### Measurement of mitochondrial membrane potential (Δψ)

JC-1 is a cationic dye that exhibits potential-dependent accumulation in mitochondria, indicated by a fluorescence emission shift from green (525 nm) to red (590 nm). Consequently, mitochondrial depolarization is indicated by a decrease in the red/green fluorescence intensity ratio. The potential sensitive color shift is due to the concentration-dependent formation of red fluorescent J-aggregates. H9c2 cells in different treatments were exposed to JC-1 staining solution (10 μg/mL) for 20 min at 37°C. The cells were then washed twice with phosphate-buffered saline (PBS) and incubated in DMEM for observation using a confocal microscope (Carl Zeiss). The ratio of red to green fluorescence intensity was analyzed as Δψ.

### Assessment of intracellular ROS

Intracellular ROS were assessed by DCFH-DA, a cell-permeable non-fluorescent probe which is de-esterified intracellularly and turns to highly fluorescent 2′,7′-dichlorofluorescein upon oxidation. At the end of reperfusion, H9c2 cells were loaded with DCFH-DA (5 μM) in DMEM for 30 min at 37°C. The cells were then washed three times with PBS. The DCF fluorescence intensity was measured using confocal microscopy at 488 excitation and 520 nm emission.

### Detection of apoptosis by DAPI staining

Apoptotic cells were evaluated using the DNA-binding fluorescent dye DAPI. The percentage of nuclear condensation (NCI) in cells represents the extent of IR injury. H9c2 cells were fixed with 4% paraformaldehyde for 20 min at room temperature and then incubated with DAPI (10 μM) dissolved in PBS for 7 min. After washing several times with PBS, fluorescence was visualized by confocal microscopy. The ratio of NCI cells in each group was counted using Image-Pro software.

### TUNEL assay

Cells from different treatment groups were fixed and immersed in 1% Triton X-100 for 5 min. After washing several times with PBS, cells were incubated in a total TUNEL reaction buffer for 1 h at 37°C. Then a Streptavidin-Fluorescein Labeling buffer was replaced for another 30 min in the same condition. Finally, the nuclei was counterstained with DAPI. The percent of apoptosis cells were decided by TUNEL positive cells (green fluorescence nuclei) to the total DAPI staining cells (blue fluorescence nuclei). Images were captured by fluorescence microscope and six random fields were calculated for apoptosis index.

### Determination of mitochondrial morphology

H9c2 cells were seeded at 3 × 10^4^/well in a 35 mm glass bottom culture dish. Following different treatments, the cells were loaded with JC-1 as previously described. Mitochondrial morphology was then determined using a confocal microscope equipped with a 100 × oil immersion objective.

### Immunoblotting

Proteins were separated by sodium dodecyl sulfate-polyacrylamide gel electrophoresis and trans-ferred onto polyvinylidene difluoride membranes (Millipore). The membranes were blocked with Tris-buffered saline containing 0.2% Tween 20 and 5% fat-free dry milk. The membranes were then incubated sequentially with primary antibodies and then horseradish peroxidase-conjugated secondary antibodies. Specific proteins were detected with the enhanced chemiluminescence detection reagent (Pierce), according to the manufacturer's protocol.

### Immunofluorescence microscopy

Cells cultured on chamber slides were fixed with 4% paraformaldehyde for 30 min, permeated in 0.5% Triton X-100/PBS for 20 min and blocked in 2% bovine serine albumin in PBS for 30 min at room temperature. The cells were then incubated in succession with primary antibodies and fluorescein- or rhodamine-conjugated secondary antibodies followed by staining with DAPI for 5 min. Coverslips were mounted with 90% glycerol in PBS and examined with a confocal microscope.

### Statistical analysis

Results are shown as mean ± standard error of the mean. Statistical analysis among different groups was assessed by one-way analysis of variance with Tukey's *post hoc* test. A *P* value < 0.05 was defined as statistically significant.

## CONCLUSIONS

In summary, our results indicate that STVNa inhibits IR-induced H9c2 myocardial cell apoptosis by down-regulating the mitochondria fission proteins, Drp1 and Fis1, recovering Δψ and decreasing intracellular ROS overproduction. STVNa may be a promising drug for the treatment of cardiovascular diseases in the future.
